# Supplementary material for: Drug Combinations Targeting FAK and MEK Overcomes Tumor Heterogeneity in Glioblastoma
Source: Pharmaceutics. 2025 Apr 23;17(5):549. doi: 10.3390/pharmaceutics17050549 (PMC12114623; doi:10.3390/pharmaceutics17050549)
Supplement: Supplementary file 1 [file pharmaceutics-17-00549-s001.zip › pharmaceutics-3542915-supplementary.pdf]

SUPPLEMENTRAY INFORMATION

Drug Combinations Targeting FAK and MEK Overcomes Tumor Heterogeneity in Glioblastoma

Muhammad Furqan [1,2,†], Richard J. R. Elliott [1,2,†], Peter W. K. Nagle [1,2], John C. Dawson [1,2], Roza Masalmeh [1,2], Virginia Alvarez Garcia [1,2], Alison F. Munro [1,2], Camilla Drake [1,2], Gillian M. Morrison [2,3], Steven M. Pollard [2,3], Daniel Ebner [4], Valerie G. Brunton [1,2], Margaret C. Frame [1,2] and Neil O. Carragher [1,2,*]

[1] Edinburgh Cancer Research, Institute of Genetics and Cancer, Western General Hospital, University of Edinburgh, Edinburgh EH4 2XU, UK
[2] Cancer Research UK Scotland Centre, University of Edinburgh, Edinburgh EH4 2XR, UK
[3] Centre for Regenerative Medicine, Institute of Regeneration and Repair, University of Edinburgh, Edinburgh EH16 4UU, UK
[4] Nuffield Department of Medicine, University of Oxford, Old Road Campus, Oxford OX3 7BN, UK
***** Correspondence: n.carragher@ed.ac.uk; Tel: +44-(0)131-651-8671-23
[†] These authors contributed equally.

Supplementary Figure S1.

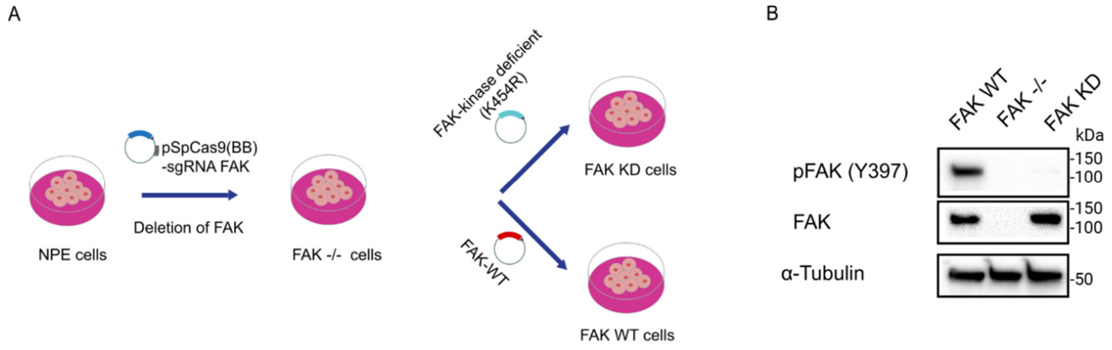

Supplementary Figure S1: Generation of FAK kinase deficient NPE cells. A. Schematic of strategy used to generate FAK_kinase deficient (K454R) cells (FAK KD) from NPE cells. B. Immunoblot of FAK WT, FAK -/- and FAK KD cells showing pFAK (Y397), and FAK expression. α-Tubulin was used as a loading control.

Supplementary Figure S2:

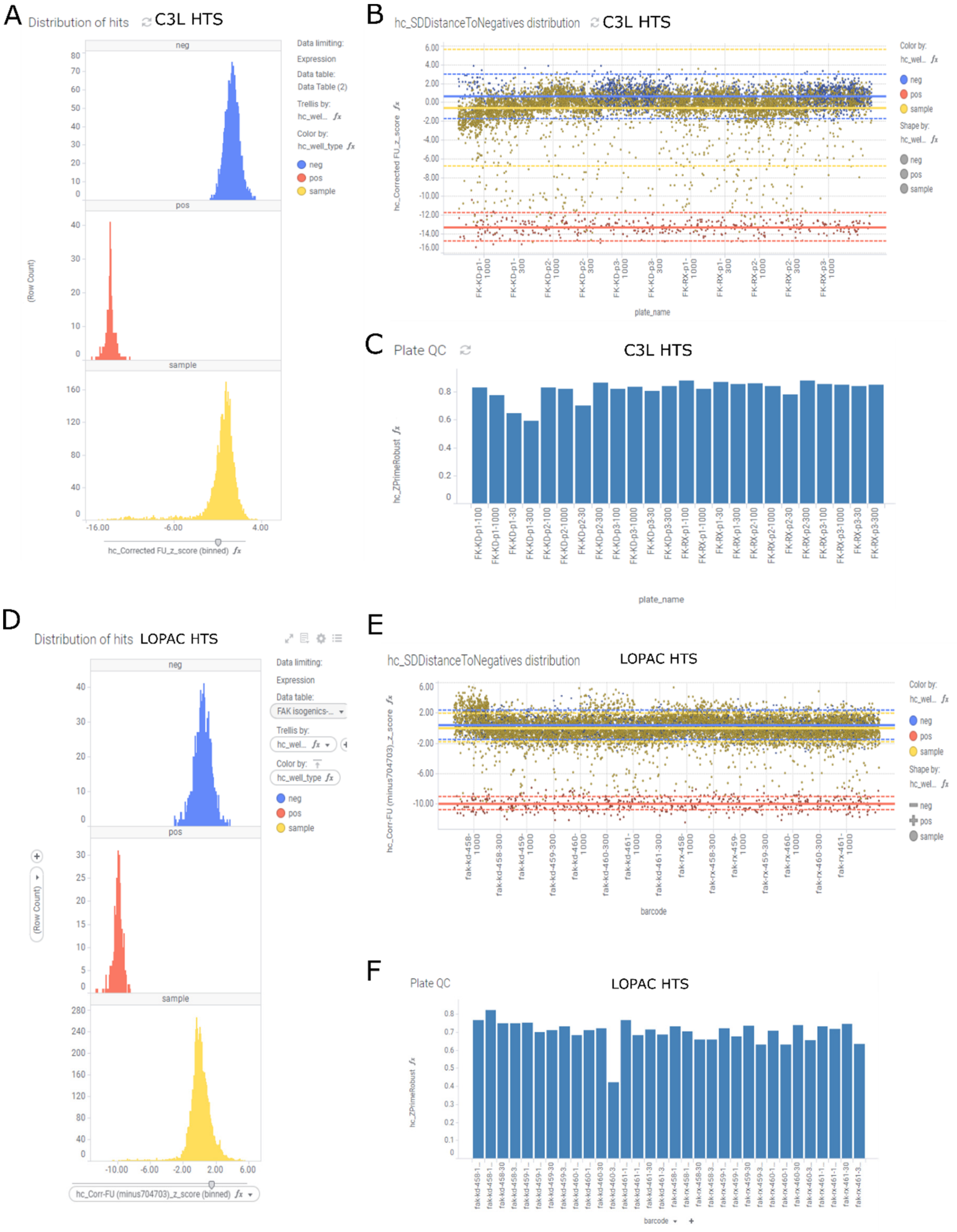

Supplementary Figure S2: Representative data of normalised high throughput screening analysis. A. C3L Library Normalised frequency distribution (Z-score) of negative controls (blue, 0.1% DMSO), positive controls (red, 1uM staurosporine) and samples (yellow). B. C3L Scatter plot representation of normalised data (Z-score) across each plate. Distribution lines (solid) represent median z-score with dispersal range (dotted lines; 3 MAD 'median-absolute deviation') for negative controls (blue, 0.1% DMSO), positive controls (red, 1uM staurosporine) and samples (yellow). C . C3L Z-prime data across each plate, ideally >0.5 for excellent assay quality. D. LOPAC Library Normalised frequency distribution (Z-score) of negative controls (blue, 0.1% DMSO), positive controls (red, 1uM staurosporine) and samples (yellow). E. LOPAC Scatter plot representation of normalised data (Z-score) across each plate. Distribution lines (solid) represent median z-score with dispersal range (dotted lines; 3 MAD 'median-absolute deviation') for negative controls (blue, 0.1% DMSO), positive controls (red, 1uM staurosporine) and samples (yellow). F . LOPAC Z-prime data across each plate, ideally >0.5 for excellent assay quality.

Supplementary Figure S2 (cont'd):

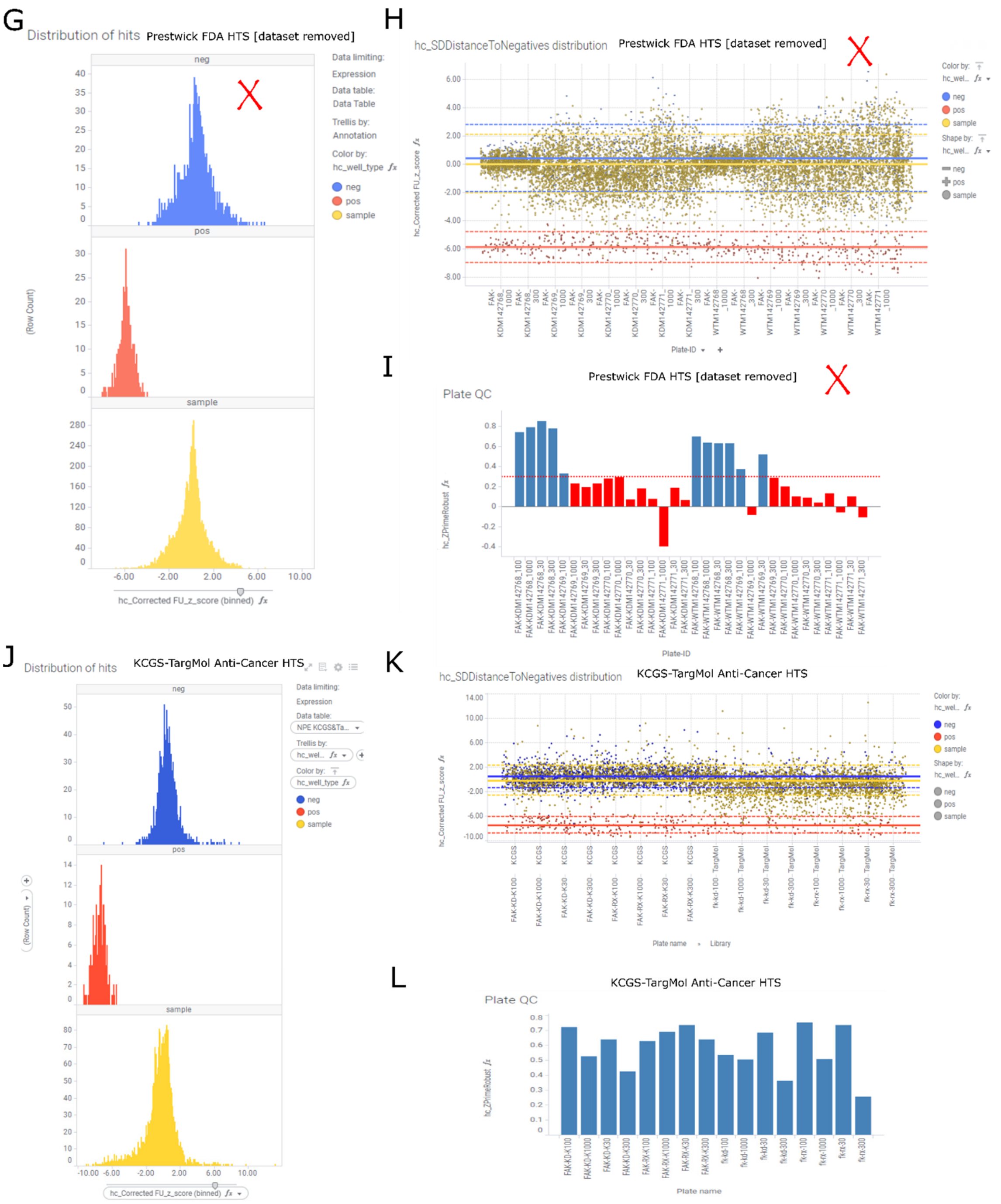

Supplementary Figure S2 cont'd : Representative data of normalised high throughput screening analysis. G. Failed QC. Data rejected. Prestwick FDA Library Normalised frequency distribution (Z-score) of negative controls (blue, 0.1% DMSO), positive controls (red, 1uM staurosporine) and samples (yellow). H. Failed QC. Data rejected. Prestwick FDA Scatter plot representation of normalised data (Z-score) across each plate. Distribution lines (solid) represent median z-score with dispersal range (dotted lines; 3 MAD 'median-absolute deviation') for negative controls (blue, 0.1% DMSO), positive controls (red, 1uM staurosporine) and samples (yellow). I. Failed QC. Data rejected. Prestwick FDA Z-prime data across each plate, ideally >0.5 for excellent assay quality. J. KCGS-TargMol Anti-Cancer Library Normalised frequency distribution (Z-score) of negative controls (blue, 0.1% DMSO), positive controls (red, 1uM staurosporine) and samples (yellow). K. KCGS-TargMol Anti-Cancer Library Scatter plot representation of normalised data (Z-score) across each plate. Distribution lines (solid) represent median z-score with dispersal range (dotted lines; 3 MAD 'median-absolute deviation') for negative controls (blue, 0.1% DMSO), positive controls (red, 1uM staurosporine) and samples (yellow). L . KCGS-TargMol Anti-Cancer Library Z-prime data across each plate, ideally >0.5 for excellent assay quality.

Supplementary Figure S3:

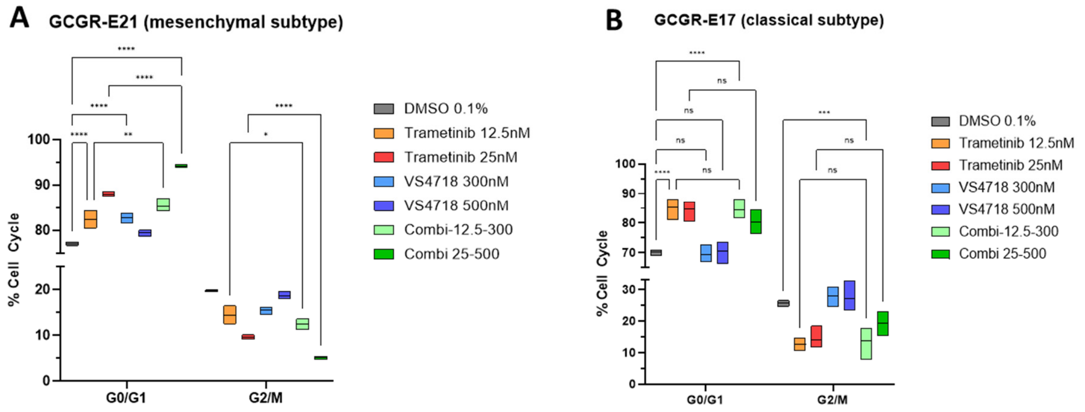

Supplementary Figure S3: Cell cycle analysis of E21 (A) E17 cells (B) [from Hoechst-33342 nuclear staining, DNA content analysis, MetaXpress Software]. Statistical analysis (Two-Way Anova) was carried out using Prism GraphPad. E21 cells showed significantly enhanced G_0/G_1 arrest in the combination relative to drug alone.

Supplementary Figure S4:

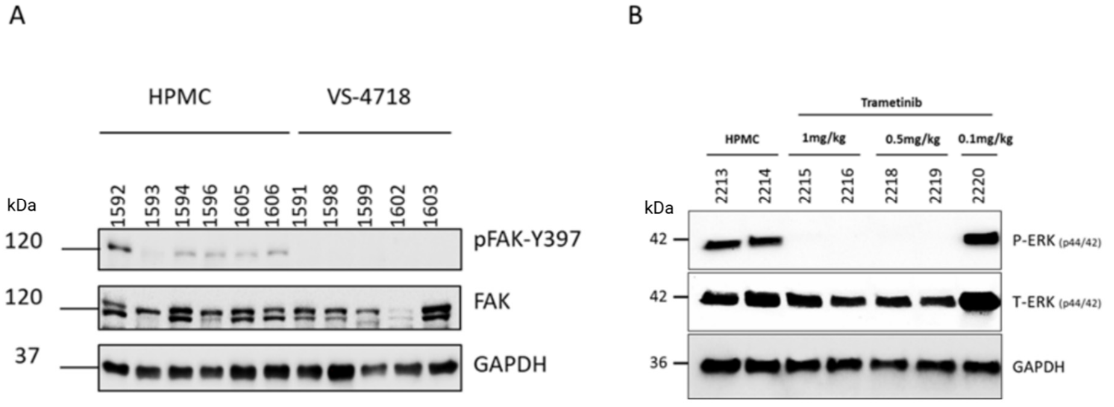

Supplementary Figure S4: In vivo biomarker modulation by FAK and MEK inhibitors reflect target engagement in mice brains. Western blot analysis of tumour lysates showing inhibition of (A) P-FAK in VS4718 75mg/kg treated tumours and (B) P-ERK in Trametinib treated tumours.

Supplementary Figure S5:

NPE-FAK-WT mice weights

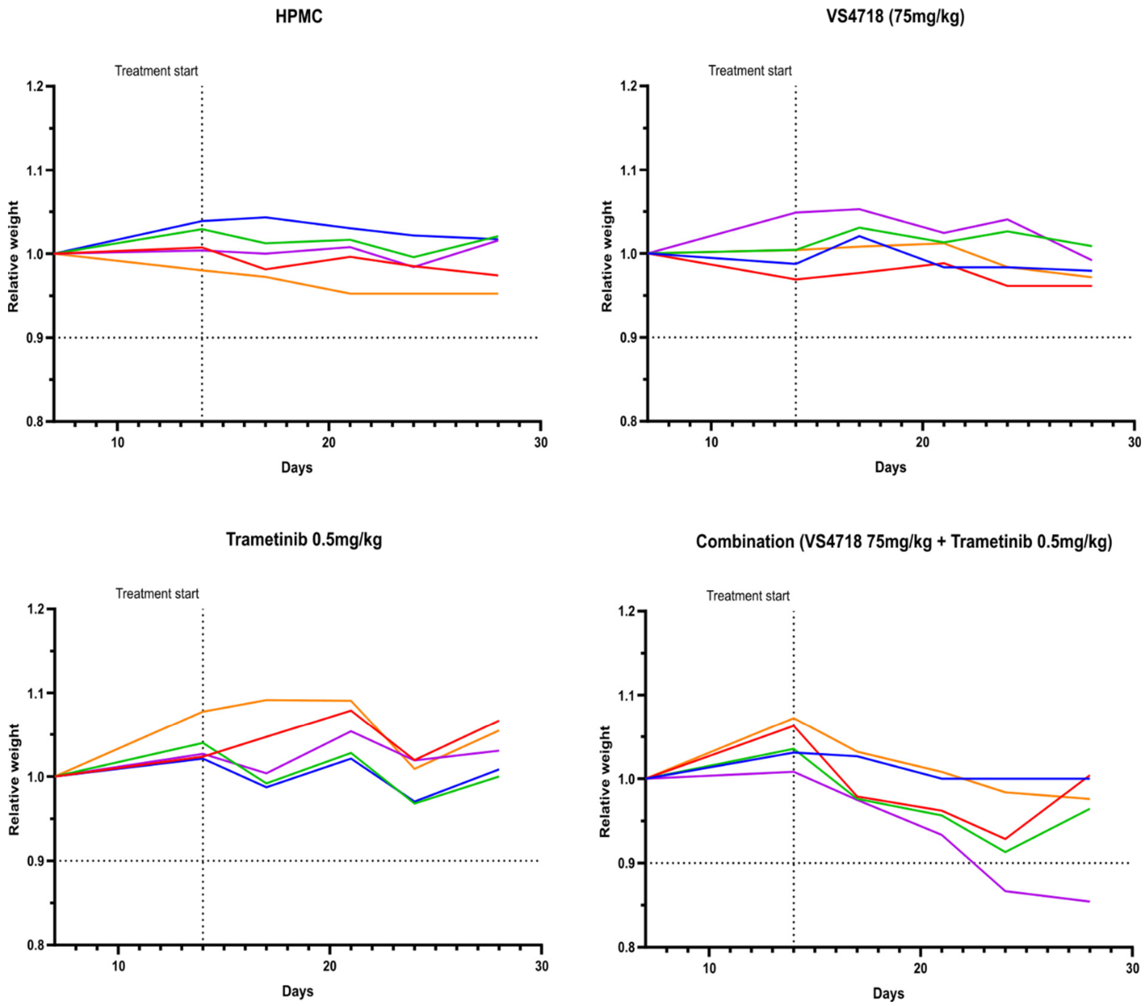

G7 mice weights

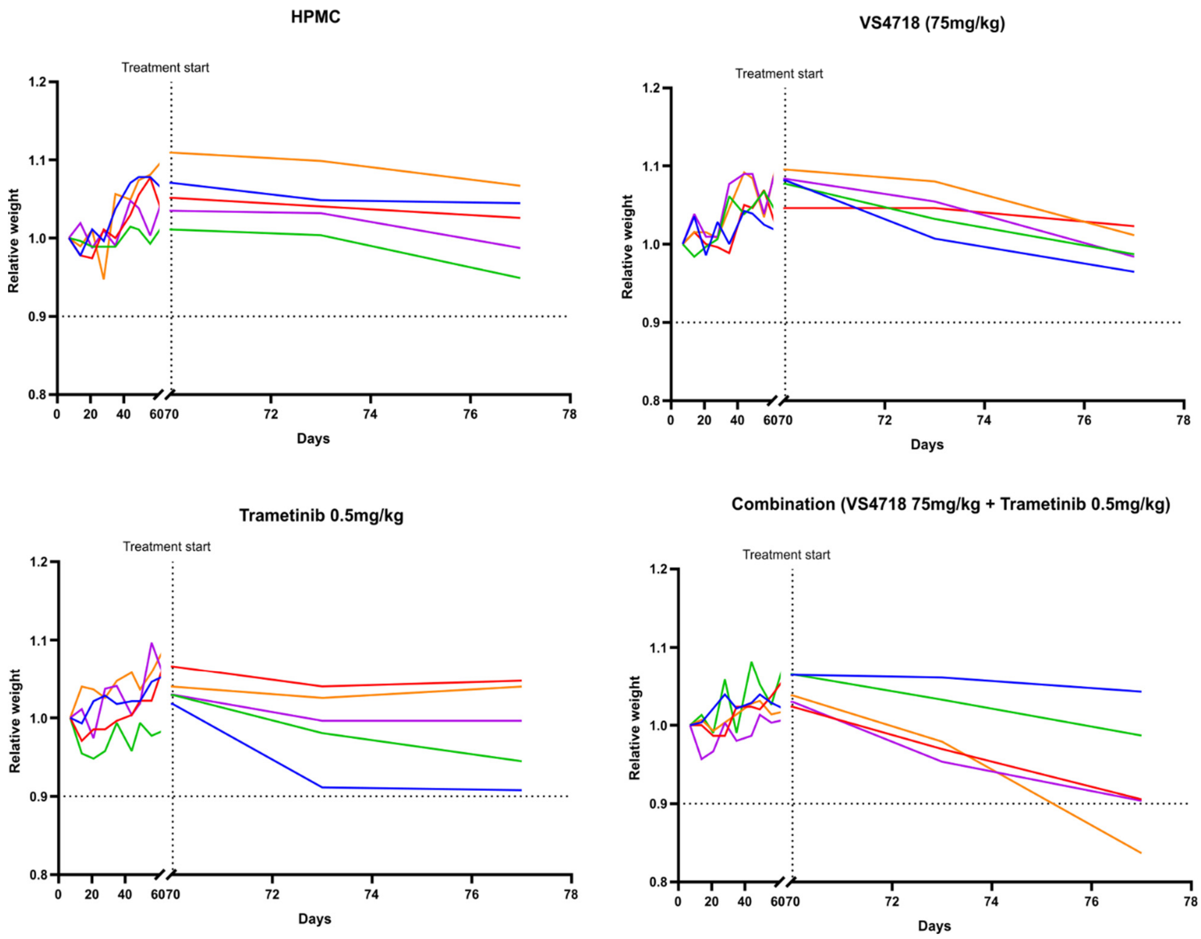

Supplementary Figure S5: Body weight measurements of NPE-FAK-WT and G7 tumour bearing mice treated with the indicated drugs over the course of two and one week, respectively via oral gavage. Relative weight was calculated by dividing the weight of each mouse on a given day by its weight on day 1. Each coloured line represents one mouse.

Supplementary Figure S6:

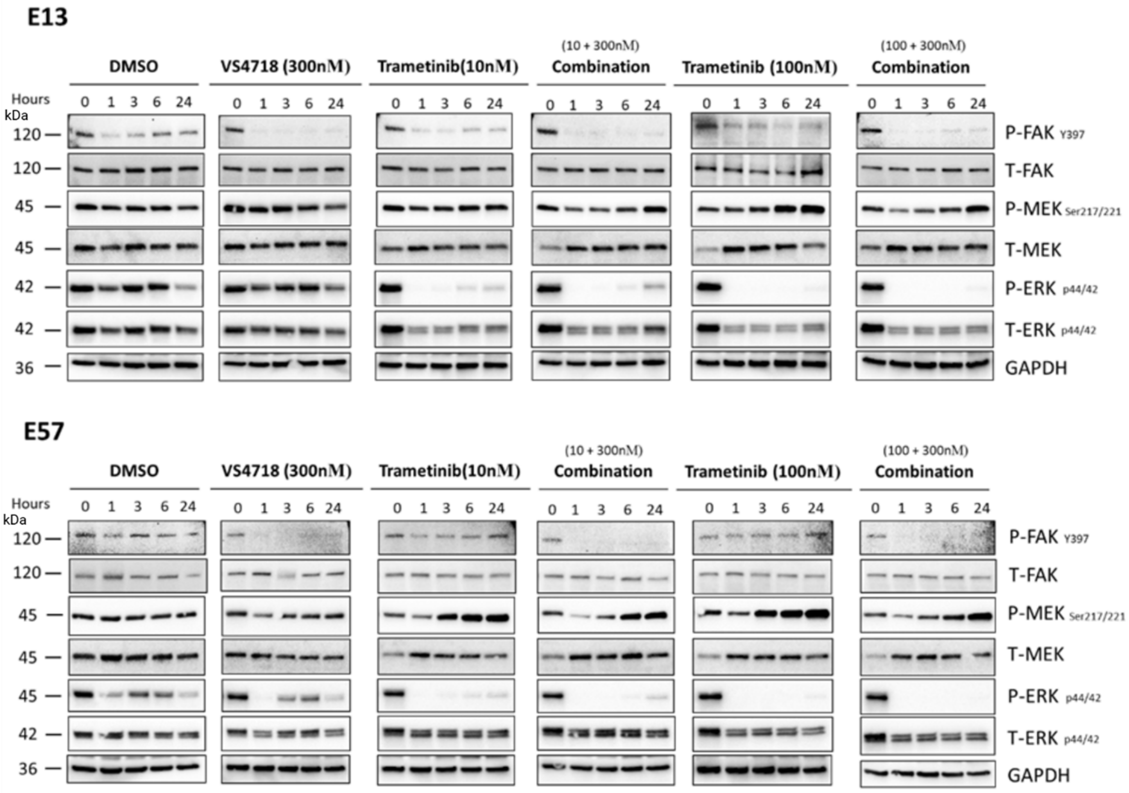

Supplementary Figure S6: Examining the time course of FAK, MEK and ERK Phosphorylation following treatment with indicated drugs in E13 and E57 cells

Supplementary Figure S7.

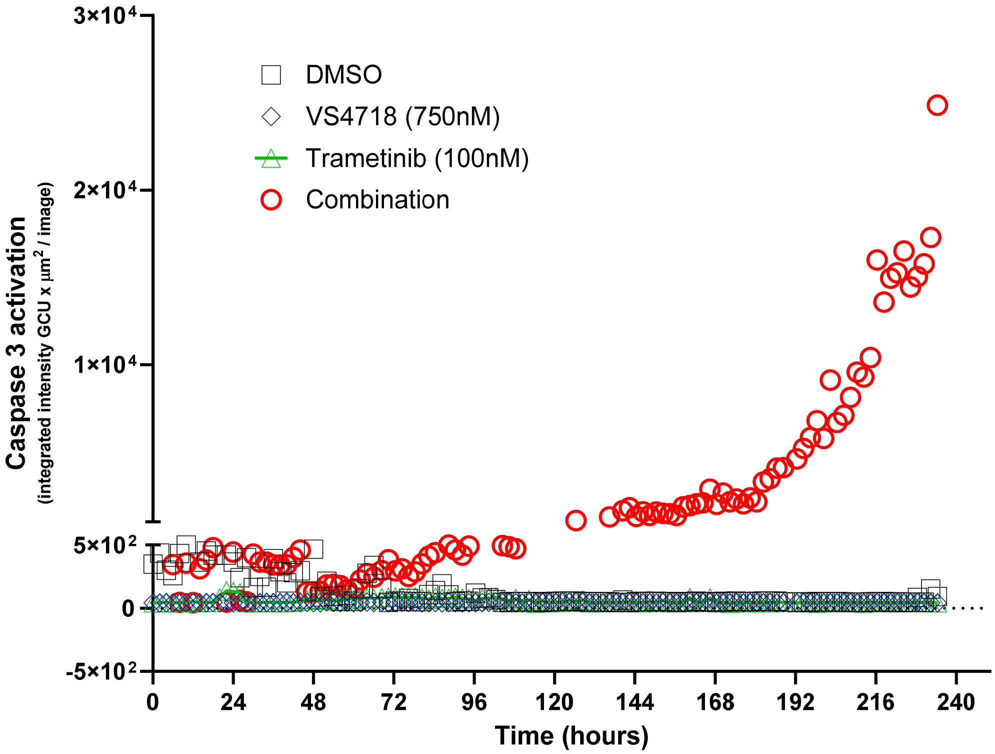

Supplementary Figure S7: Treatment with FAK-MEK inhibitors combination causes activation of caspase-3 in G7 GBM spheroids. Caspase-3 activation was determined using BioTrac 530 Red Caspase-3 Dye (Sigma-Aldrich) according to manufacturer's instructions.

Supplementary Figure S8.

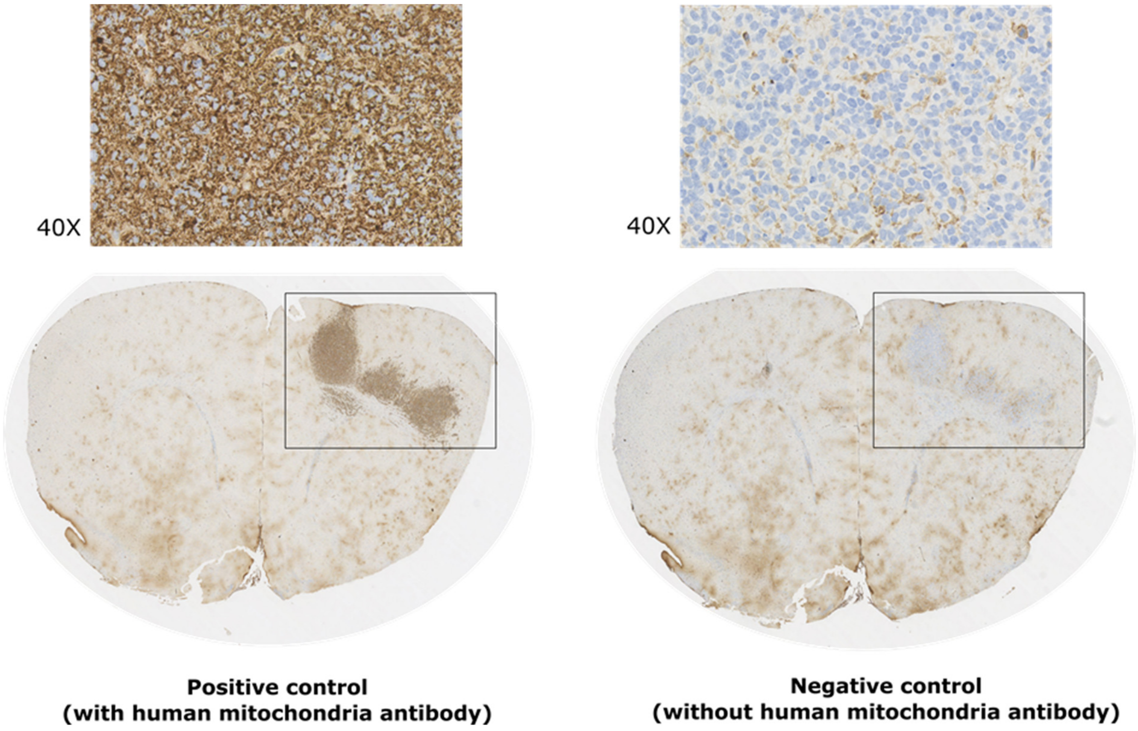

Supplementary Figure S8: Immunohistochemistry using an anti-mitochondria antibody with positive and negative controls to assess specificity. The positive control (left) shows strong mitochondrial staining, while the negative control (right) displays minimal non-specific binding. Boxed regions highlight staining patterns for comparison.
